# Association between fear states and cognitive impairment: A cross-sectional observational study based on the China Health and Retirement Longitudinal Study (CHARLS)

**DOI:** 10.1097/MD.0000000000050673

**Published:** 2026-09-11

**Authors:** Xiaoyun Hui, Kaikai Jiang, Jing Ji, Chunxuan Dong, Xiumei Xu

**Affiliations:** a Department of Rehabilitation Medicine, Wuwei People’s Hospital, Wuwei, Gansu, 733099, China; b Tianshui Maternal and Child Health Hospital, Tianshui, Gansu, 741000, China; c Department of Rehabilitation Medicine, Gansu Provincial Hospital of Traditional Chinese Medicine, Lanzhou, Gansu, 730050, China.

**Keywords:** Anxiety, China Health and Retirement Longitudinal Study, Cognitive dysfunction, Fear, Observational study

## Abstract

As global populations age, the prevalence of cognitive impairment continues to rise, increasing the burden on families and society. This study examined the relationship between anxiety and cognitive function. Using nationally representative data from China Health and Retirement Longitudinal Study (CHARLS), this observational cohort study evaluated the association between fear states and cognitive impairment to provide objective scientific evidence on the potential causes of cognitive decline and to inform early prevention and treatment strategies. In this study, the prevalence of cognitive impairment was significantly higher among participants in the fearful group. After adjustment for multiple covariates, fearful state remained independently associated with cognitive impairment (odds ratio = 2.38, 95% confidence interval = 1.24–4.59, *P* = 9.52 × 10^−3^). Smooth curve fitting additionally confirmed this positive association. These findings demonstrate a significant association between fear state and cognitive impairment, highlighting the need to consider emotional well-being in cognitive health assessments.

## 1. Introduction

Cognitive impairment (CI) is a neurological condition marked by a substantial decline in cognitive ability, involving deficits in one or more key brain functions (such as memory, learning, attention, and decision-making).^[1]^ CI is prevalent among older adults, diminishing quality of life and imposing a major burden on families and society.^[2]^ Demographic shifts have led to a steady global increase in the elderly population, resulting in a higher prevalence of CI.^[3]^ The World Health Organization projects that by 2030, one in six individuals worldwide will be aged ≥ 60 years. The population aged > 60 years is expected to increase from 1 billion in 2020 to 1.4 billion by 2030. Furthermore, by 2050, this group is projected to double, reaching 2.1 billion. Additionally, the population aged ≥ 80 years is estimated to triple, reaching 426 million between 2020 and 2050.^[4]^ The primary independent risk factor for CI is advanced age,^[5]^ with risk rising markedly with age. Vascular risk factors also elevate the risk of CI, and stroke increases CI risk and severity.^[6]^ Moreover, individuals with chronic kidney disease face a substantially higher risk of developing CI compared with the general population.^[1]^ Previous studies indicate that several variables, including age, educational attainment, diabetes duration, fasting blood glucose levels, HbA1c content, total cholesterol levels, triglyceride content, and 24-h urinary protein excretion, are independent risk factors for CI in elderly patients with type 2 diabetes mellitus.^[7]^ In addition to these factors, prior research suggests that neuroinflammation plays a key role in CI pathogenesis. Systemic inflammatory responses can activate central nervous system inflammatory pathways, leading to neuronal damage, synaptic dysfunction, and neurotransmitter imbalance, thereby accelerating cognitive decline.^[8,9]^ For example, a randomized controlled trial involving patients undergoing cardiac surgery showed that preoperative dexamethasone (an anti-inflammatory drug) significantly reduced the risk of early postoperative cognitive impairment while decreasing the levels of inflammatory markers, such as C-reactive protein, indicating that inflammatory responses are key mechanisms underlying CI, particularly under acute stress or trauma situations.^[10]^ These findings deepen our understanding of CI’s multifactorial mechanisms and imply that emotional and stress states (such as fear) influence cognitive function by modulating inflammation. Therefore, early identification of cognitive decline–associated factors is essential for developing effective prevention and intervention strategies. This will not only improve quality of life in older adults but also reduce societal and familial burdens and better address the challenges of population aging.

Fear usually refers to an individual’s strong emotional response to perceived threats or danger. The physical and psychological features of fear include increased heart rate, sweating, heightened alertness, and related responses. Fear can also trigger anxiety and stress.^[11]^ Prior neuroimaging studies in humans have primarily focused on the “fear network,” which includes key brain regions such as the ventromedial prefrontal cortex, dorsal anterior cingulate cortex, insular cortex, hippocampus, and amygdala. These regions play essential roles in modulating fear, arousal, and threat detection, and the regulation of responses to both fear and conditioned stimuli.^[12]^ Evidence from attention bias research indicates that anxiety alters attentional function, although findings remain inconsistent.^[13]^ Depression and anxiety are prevalent among patients with dementia. A systematic review and meta-analysis reported that the prevalence of anxiety disorders in mild, moderate, and severe dementia was 38% (95% confidence interval: 31–45%), 41% (95% confidence interval: 31–52%), and 37% (95% confidence interval: 8–82%), respectively, and anxiety in vascular dementia was higher in Europe than in Asia.^[14]^ These findings indicate that anxiety is closely linked to cognitive function, highlighting the need to explore interventions that reduce fear and slow cognitive decline. In addition, previous studies^[15]^ have shown that fear of negative evaluation is a risk factor for eating disorders, and higher trait levels of negative evaluation increase the likelihood of restrictive eating behaviors. CI often occurs after traumatic brain injury. Notably, memory functions are particularly vulnerable to decline during recovery from long-term injuries.^[16]^ Fear-related conditions, such as post-traumatic stress disorder, may further increase the risk of aged-related cognitive decline.^[17]^ Moreover, fear and avoidance of dementia symptoms in older adults can lead to adverse outcomes, including reduced quality of life, poor physical health, and memory loss, thereby accelerating cognitive decline.^[18]^ Related studies^[19]^ have indicated that a strong fear of falling increases the risk of cognitive decline in older individuals. Overall, fear states appear to play an important role in the development of CI, although the precise relationship remains unclear. Therefore, the present study examines the association between fear state and CI.

The China Health and Retirement Longitudinal Study (CHARLS) is a large national development research institute sponsored by the Beijing interdisciplinary longitudinal study of the database. The survey uses a stratified, multistage probability-proportional sampling method to assess individuals aged ≥ 45 years across 28 provinces, including autonomous regions and municipalities directly under the central government. This survey collects data on demographics, family background, health and function, healthcare access and insurance, income, employment status, and asset ownership.^[20]^ The national baseline survey was conducted in 2011 and has been followed up every 2–3 years in 2013, 2015, 2018, and 2020 to examine associations among diseases and related risk factors. Using CHARLS data, Lin et al^[21]^ revealed that depression symptoms in middle-aged and older adults with chronic diseases are influenced by multiple factors. Additionally, Jiang et al^[22]^ used CHARLS data to show that economic intergenerational support has no major effect on older adults’ psychological health, whereas emotional support and daily care reduce depression levels and are associated with improved mental health. Therefore, the CHARLS database provides a valuable and robust resource for research and has become an essential tool in studies of middle-aged and elderly populations.

In this context, the present study explores the association between fear state and CI using nationally representative CHARLS data. We propose the following hypothesis: among individuals aged ≥ 45 years, a significant positive correlation exists between self-reported fear state and CI. We test the independence and robustness of this association using multivariate statistical models, adjusting for a range of demographic and health-related covariates, to provide new evidence for understanding factors associated with CI.

## 2. Materials and methods

### 2.1. Data collection

This observational cohort study used data from CHARLS (https://charls.pku.edu.cn/en/). In the present study, all participants were drawn from the 2018 wave, based on questionnaire data for individuals with CI aged ≥ 45 years (n = 19,744). First, individuals with a history of memory-related disease were excluded. In addition, participants with missing data on various variables, including sex, medical insurance, stroke, diabetes, cancer or malignant tumors, heart attack, hearing problems, health status, social activity, fear status, Mini-Mental State Examination (MMSE) scores, and education level, were excluded. Ultimately, 2001 participants were included in the cohort analysis. The detailed participant selection process is shown in **STROBE Flow Diagram**. This study was a secondary analysis of a publicly available database and did not involve new recruitment, interventions, or biospecimen collection. The CHARLS database provides anonymized data, and ethical approval and informed consent were obtained for all primary data collection; therefore, no additional ethical approval was required for the present study. This observational cohort study was reported in accordance with the STrengthening the Reporting of OBservational studies in Epidemiology (STROBE) statement guidelines.

### 2.2. CI assessment

CI was assessed using the Chinese version of the 2018 MMSE questionnaire, which includes 24 items across 4 cognitive domains. MMSE scores ranged from 0 to 30, with lower scores indicating poorer cognitive function. Cognitive status was categorized by education level: illiteracy < 17, primary school < 20, secondary education or greater < 24. Participants with scores < 24 were classified as having CI, whereas others were categorized as non-CI (normal group).^[23]^

### 2.3. Exposure assessment

Fear status was determined based on the questionnaire item dc014: “Have you ever felt fearful?” Participants who answered “sometimes or half the time” or “most of the time” were classified as the fearful group. Those who responded “rarely or not at all” or “not too much” served as the normal group.

### 2.4. Covariates

Additional covariates included sex (ba000_w2_3; male or female), medical insurance (ea001_w4_s12; yes or no), stroke (da007_8; yes or no), diabetes (da007_3; yes or no), cancer or malignant tumors (da007_4; yes or no), heart attack (da007_7; yes or no), hearing problems (da005_4; yes or no), health status (da002; good, fair, or bad), and activities (da056_s12; yes or no).

### 2.5. Statistical analysis of participant characteristics

All participants from the CHARLS database were divided into 2 groups based on the presence or absence of CI. Differences in baseline characteristics (sex, medical insurance, stroke, diabetes, cancer or malignant tumors, heart attack, hearing problems, health status, activities, and fear status) between groups were analyzed using a weighted chi-square test, with significance set at *P* < .05.

### 2.6. Statistical analysis

To assess multicollinearity between fear status and other covariates, variance inflation factors were calculated using the car package (v3.1.1; https://socialsciences.mcmaster.ca/jfox/Books/Companion/). Because some covariates were categorical with multiple levels, the generalized variance inflation factor (GVIF) and its standardized form GVIF^1/(2 × Df)^ were adopted for interpretation; values closer to 1 and well below 5 indicated no significant multicollinearity.

Within a complex sampling weighting framework, a survey design was constructed using sampling weights and clustered primary sampling units. Three weighted generalized linear models (GLMs) were fitted using the survey (v4.2.1) package (https://cran.r-project.org/web//packages/survey/survey.pdf) to evaluate the association between fear status and CI. Model I (unadjusted) included fear status only; Model II (minimally adjusted) also included sex and medical insurance; Model III was further adjusted for stroke, diabetes, cancer or malignant neoplasms, heart disease, hearing problems, health status, activities, and memory-related disorders. Adjusted odds ratios (OR) and 95% confidence intervals were calculated. The complex sampling design-based Wald F test was used to assess the overall significance of the GLMs. To evaluate calibration and model fit for Model III, weighted decile calibration curves were plotted, and calibration regression (observed outcome regressed on the logit of predicted probability) was performed. Deviance residual diagnostics were also conducted to assess model fit and identify potential outliers or influential observations. To further assess robustness, the nonlinear relationship between fear status and CI in Model III was examined using a smooth curve.

To evaluate the relative contribution of predictors, eXtreme Gradient Boosting (XGBoost; binary classification, binary:logistic) was used to assess feature importance. Data were split into training and test sets at an 80:20 ratio; fivefold stratified cross-validation was applied for hyperparameter tuning in the training set, and early stopping was used to prevent overfitting and determine the optimal number of iterations. The final model employed optimized hyperparameters: max_depth = 3, eta = 0.05, min_child_weight = 1, subsample = 0.9, colsample_bytree = 0.7, gamma = 1, with L2 regularization (lambda = 1, alpha = 0), and the optimal number of iterations (best_nrounds = 28). Feature importance rankings based on Gain and corresponding bar plots were generated to illustrate predictor contributions.

Subgroup analyses were conducted based on Model III to examine whether the association between fear status and CI varied across population subgroups. Owing to unstable model convergence caused by limited sample size, event counts, or complete separation in some subgroups, analyses were restricted to sex, activities, hearing problems, and heart attack.

All statistical analyses were performed in R (v4.2.2). Continuous variables were expressed as mean ± standard deviation. Student *t* test was used to compare differences between 2 groups.

## 3. Results

### 3.1. Differences in baseline characteristics

After stratifying participants by CI status, they were categorized into CI and normal groups. As shown in Table 1, fear status showed a significant association with CI (*P* = .035). Specifically, 14 participants were both fearful and had CI, 110 were fearful without CI, 114 were not fearful but had CI, and 1763 were neither fearful nor had CI (Table 1). Additionally, medical insurance and activities differed significantly between participants with and without CI (*P* < .05). Results from the multicollinearity analysis revealed that GVIF and GVIF^1/(2Df)^ values for all variables were close to 1 and well below 5, indicating no major multicollinearity (Table 2).

**Table 1:** Effects of participant characteristics on cognitive impairment.

	level	No	Yes	*P*
n		1873	128	
Fearful (%)	No	1763 (94.1)	114 (89.1)	.035
	Yes	110 (5.9)	14 (10.9)	
Gender (%)	Male	1267 (67.6)	83 (64.8)	.577
	Female	606 (32.4)	45 (35.2)	
Medical_insurance (%)	Yes	1851 (98.8)	121 (94.5)	<.001
	No	22 (1.2)	7 (5.5)	
Stroke (%)	Yes	86 (4.6)	7 (5.5)	.811
	No	1787 (95.4)	121 (94.5)	
Diabetes (%)	Yes	96 (5.1)	8 (6.2)	.727
	No	1777 (94.9)	120 (93.8)	
Cancer_or_malignant_tumor (%)	Yes	27 (1.4)	1 (0.8)	.821
	No	1846 (98.6)	127 (99.2)	
Heart_attack (%)	Yes	166 (8.9)	7 (5.5)	.246
	No	1707 (91.1)	121 (94.5)	
Hearing_problem (%)	Yes	99 (5.3)	8 (6.2)	.79
	No	1774 (94.7)	120 (93.8)	
Healthy_status (%)	Good	533 (28.5)	35 (27.3)	.945
	Fair	1035 (55.3)	71 (55.5)	
	Poor	305 (16.3)	22 (17.2)	
Activities (%)	Yes	1100 (58.7)	55 (43.0)	.001
	No	773 (41.3)	73 (57.0)	

**Table 2:** Multicollinearity assessment of variables.

Variable	GVIF	Df	GVIF^1/(2Df)^
Fearful	1.0337433	1	1.0167317
Gender	1.020781	1	1.0103371
Medical_insurance	1.0028105	1	1.0014043
Stroke	1.0190296	1	1.00947
Diabetes	1.0220784	1	1.0109789
Cancer_or_malignant_tumor	1.0275829	1	1.0136976
Heart_attack	1.0375511	1	1.0186025
Hearing_problem	1.0142806	1	1.007115
Healthy_status	1.0977641	2	1.0235929
Activities	1.0170468	1	1.0084874

### 3.2. Association between fear status and CI risk

**Figure 1.**
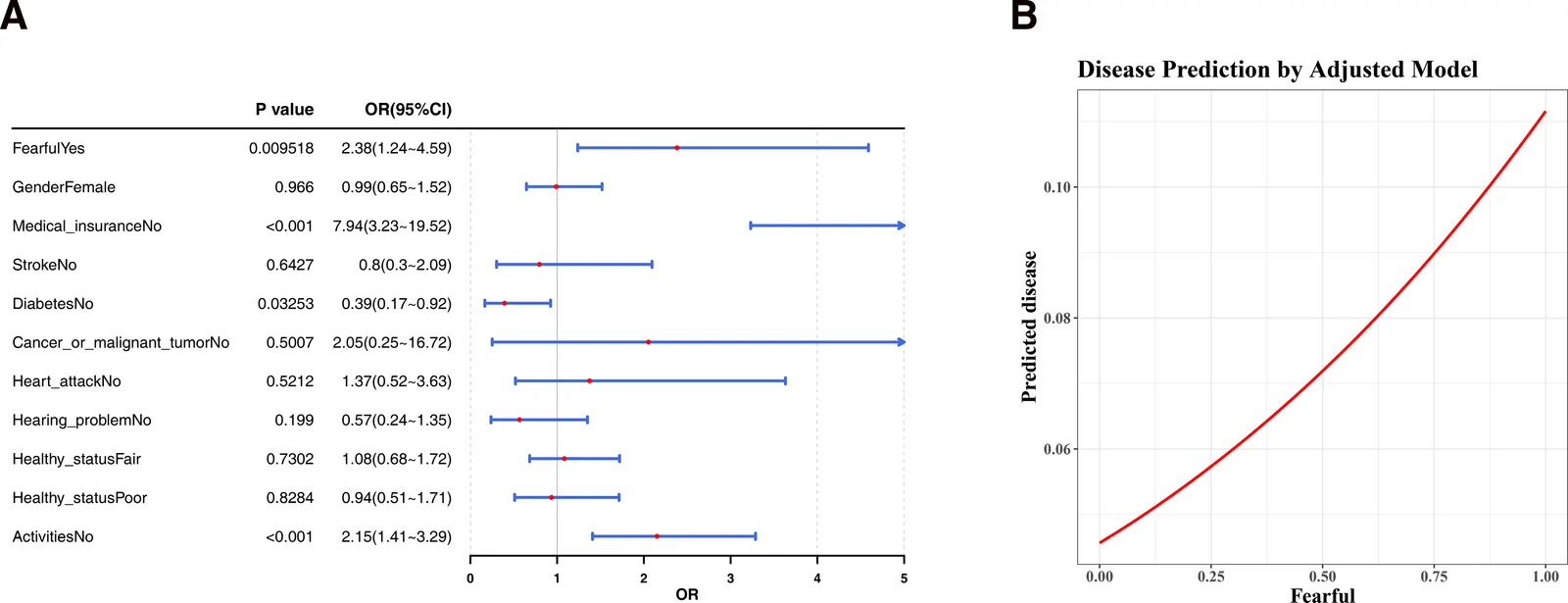
Association between fearful state and the risk of CI, (A) Forest plot of the association between fearful state and CI risk based on the Model III. The red dots in the figure represent the OR of each variable, and the horizontal lines represent the 95% confidence intervals. The dotted line represents the invalid line with OR = 1. The OR (95% CI) and Wald test *P* value are given on the right side. (B) Smooth curve fitting of the correlation between fearful state and the predicted prevalence of CI. The horizontal axis represents the Fearful state (0 = not fearful, 1 = fearful; continuous scales are used to show the trend of smooth fitting), and the vertical axis represents the probability of CI predicted by the model.

Based on three weighted GLMs, association analyses revealed that the relationship between fearfulness and CI was not significantly confounded by other covariates (Model I: *P* = 3.35 × 10^−3^, OR = 2.63, 95% confidence interval = 1.38–5.01; Model II: *P* = 4.24 × 10^−3^, OR = 2.58, 95% confidence interval = 1.35–4.92; Model III: *P* = 9.52 × 10^−3^, OR = 2.38, 95% confidence interval = 1.24–4.59; Table 3 and Fig. 1A). The Wald F joint test indicated that Models I, II, and III were statistically significant (*P* < .05; Table 4). Moreover, the calibration curve for Model III was close to the 45° reference line, with a calibration intercept ≈ 0 and calibration slope ≈ 1, indicating no evident systematic miscalibration (Fig. S1A, Supplemental Digital Content 1). Additionally, residuals from Model III showed a typical “double-band” distribution for binary classification outcomes, with no clear trend across fitted values, suggesting no major model misfitting (Fig. S1B, Supplemental Digital Content 1). Furthermore, smooth curve analysis showed a positive association between fearfulness and CI prevalence (Fig. 1B).

**Table 3:** Association analysis across 3 models.

exposure	model1_OR(95%_CI)	model2_OR(95%_CI)	model3_OR(95%_CI)
Fearful	2.45e + 00(1.38e + 00–4.12e + 00)	2.41e + 00(1.34e + 00–4.09e + 00)	2.19e + 00(1.18e + 00–3.85e + 00)
p_value	1.26E-03	1.83E-03	8.92E-03

**Table 4:** Overall significance of the 3 models.

model	F	df_num	df_den	*P*_value
model 1	8.7008965	1	431	.0033542
model 2	8.4344946	3	431	1.86E-05
model 3	4.5823997	11	431	1.37E-06

### 3.3. Importance of fear status for CI

**Figure 2.**
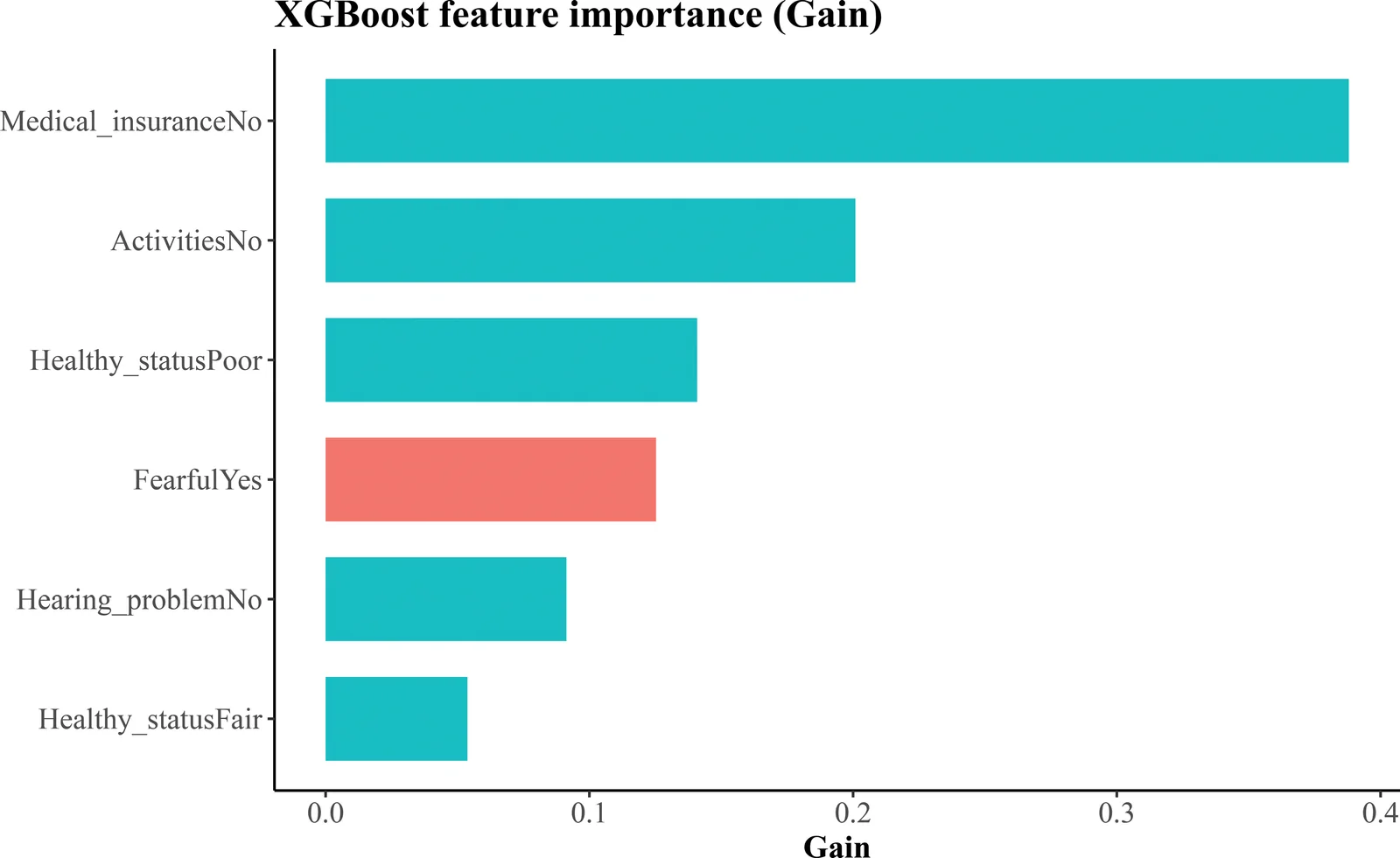
The results of covariates calculation by XGBoost algorithm.

XGBoost analysis of covariates showed that lack of medical insurance had the highest relative importance for CI, followed by lack of activities, poor health status, and fearfulness (Fig. 2). These findings suggest that fear contributes substantially to CI risk.

### 3.4. Subgroup analysis of the fear status–CI association

**Figure 3.**
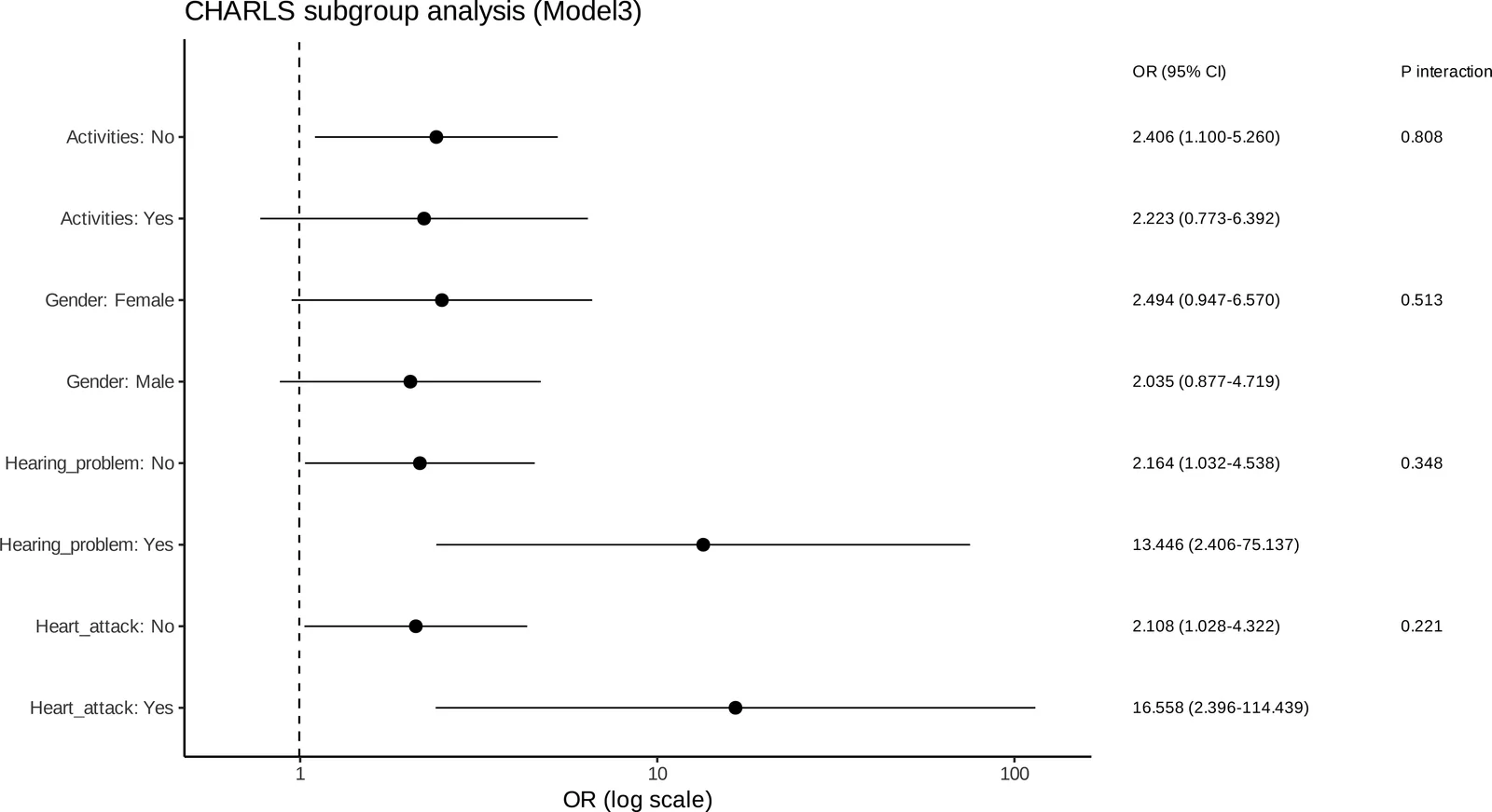
Subgroup analysis of the association between fearful state and CI risk in the CHARLS cohort (Model III).

Subgroup analyses showed that the association between fear status and CI was generally consistent across all subgroups, with no significant interaction effects (*P* > .05). Notably, participants with fearfulness who had hearing problems or a history of heart attack exhibited a higher risk of CI (OR = 13.446 and 16.558, respectively; Fig. 3).

## 4. Discussion

This study used the CHARLS database to investigate the association between fear status and CI. The results demonstrate a significant positive association between fearfulness and CI. Specifically, individuals experiencing fear had a higher risk of CI compared with those showing no fear, and this association remained significant after adjustment for multiple covariates, including medical insurance, health status, and diabetes. These findings suggest that fear may play a key role in cognitive decline.

This study revealed significant differences in medical insurance and activities between participants with and without CI (*P* < .05). Additionally, smooth curve analysis indicated a positive association between fear and CI prevalence, indicating that fear is linked to increased CI risk. Current evidence suggests that elevated fear levels are associated with changes in synaptic proteins in the amygdala, a key region regulating fear responses and negative emotions.^[24]^ Bottinelli et al.^[25]^ reported that panic attacks involve sympathetic nervous system activation and cognitive responses triggered by perceived threats. It has also been shown that baseline brain inflammatory activity, including NLRP3 in juvenile mice, is required for conditioned flexibility in the ventral hippocampus and basolateral amygdala, and that NLRP3 loss impairs synaptic transmission and promotes anxiety-like behavior and enhanced fear learning.^[26]^ Activation of the NLRP3 inflammasome has been linked to the development of various central nervous system disorders. Furthermore, NLRP3-induced and caspase-1–mediated inflammation plays a crucial role in behavioral and cognitive dysfunction in chronic brain diseases.^[27]^ Prior studies have shown that the heightened CI risk associated with fear may involve amygdala dysfunction and NLRP3 inflammasome activation.^[26]^ Overall, these findings highlight a significant association between fear and CI, showing that heightened fear states may contribute to cognitive decline.

Social avoidance is commonly associated with social anxiety disorder, characterized by fear, anxiety, or restlessness in social situations that may lead to reduced or avoided social engagement. Fear and chronic stress activate the hypothalamic–pituitary–adrenal (HPA) axis, leading to the release of stress hormones, such as cortisol, and prolonged cortisol elevation may adversely affect brain structure and function, especially in regions related to emotion and memory, such as the hippocampus.^[28]^ These mechanisms help explain how fear states affect CI.

This study revealed that the relative importance of social activities was also higher in the CI group. Social engagement may exert neuroprotective effects through several mechanisms. First, the cognitive reserve hypothesis suggests that frequent social interaction provides sustained cognitive stimulation, enhancing neural network plasticity and complexity, thereby delaying cognitive decline.^[29]^ Neuroimaging studies have shown that individuals with larger social networks possess greater gray matter volume in brain regions such as the amygdala, insula, thalamus, and frontal lobe, which are closely associated with memory, decision-making, and emotional regulation.^[29]^ Second, from a neuroendocrine perspective, social isolation activates the HPA axis, leading to chronically elevated cortisol levels. Prolonged cortisol exposure can damage limbic structures, such as the hippocampus, and accelerate cognitive decline.^[30,31]^ Furthermore, reduced social activity is associated with increased neuroinflammation; specifically, NLRP3 inflammasome activation may mediate social isolation–related cognitive impairment.^[32]^ Third, behavioral and psychological pathways indicate that social isolation is associated with depression, reduced physical activity, and accumulation of cardiovascular risk factors, which collectively contribute to cognitive impairment.^[33]^ Notably, a large-scale cohort study by Shen et al.^[34]^ using UK Biobank data reported that social isolation increases dementia risk by 26%, independent of genetic risk. Therefore, promoting social participation among older adults may serve as an important strategy for preventing CI.

### 4.1. Limitations and future research

Although our findings show a significant association between fear status and CI, the study has several limitations. First, the exposure variable “fear state” was measured using a single self-report item (dc014) and dichotomized into fear and non-fear categories. This approach did not use standardized, multidimensional psychological scales (such as the GAD-7 or STAI) and may not fully capture the frequency, intensity, or content of fear, potentially introducing measurement error and misclassification bias. Second, owing to its cross-sectional design, this study could not determine the temporal sequence or causal relationship between fear status and CI. Third, although the model was adjusted for multiple covariates, it did not account for important variables such as depressive symptoms, socioeconomic status, smoking status, alcohol consumption, and sleep quality, potentially resulting in residual confounding. Finally, the biological mechanisms linking fear status and CI remain unclear.

To address these limitations, future research should proceed in several directions. First, standardized multidimensional scales should be used to measure fear or continuous anxiety and assess severity and subtypes more accurately. Second, longitudinal cohort studies or intervention trials should be conducted to clarify temporal relationships and causality between fear status and CI. Additionally, a broader range of covariates should be incorporated to improve the robustness and generalizability of findings. Finally, integrating neuroimaging, biochemical markers (e.g., inflammatory indicators and HPA axis–related measures), and animal models may help elucidate the biological mechanisms underlying the association between fear status and CI. These improvements may provide stronger and more comprehensive evidence on the relationship between fear and CI.

## 5. Conclusions

This study identified a significant association between fear and CI. Fear states may exacerbate CI and potentially contribute to a negative feedback cycle. However, temporal relationships and causality require further investigation in longitudinal or interventional studies. Therefore, clinicians managing CI should consider assessing emotional states, and future research should clarify the direction of this association.

## Acknowledgments

This work was supported by the study based on HIF-1a regulation of iron death of hippocampal neurons, to explore the effect and mechanism of brain-buxing ointment in improving vascular dementia [grant number GZKZ-2024-1]; and the 2022 construction project of inheritance studio of Sun Qibin, national famous old Chinese medicine expert [grant number Chinese Medicine Education Letter [2022] No. 75]. The authors declare that there are no conflicts of interest. Thanks to Bioinformatics Analysis Platform of Jining Medical University for data analysis support.

## Author contributions

**Data curation:** Xiaoyun Hui.

**Investigation:** Xiaoyun Hui.

**Methodology:** Xiaoyun Hui.

**Validation:** Xiaoyun Hui.

**Formal analysis:** Kaikai Jiang.

**Resources:** Kaikai Jiang.

**Software:** Kaikai Jiang.

**Writing – original draft:** Kaikai Jiang.

**Visualization:** Xiumei Xu.

**Funding acquisition:** Chunxuan Dong, Jing Ji.

**Supervision:** Chunxuan Dong.

**Project administration:** Jing Ji.


