# Supplementary material for: Association between fear states and cognitive impairment: A cross-sectional observational study based on the China Health and Retirement Longitudinal Study (CHARLS)
Source: Medicine (Baltimore). 2026 Sep 11;105(37):e50673. doi: 10.1097/MD.0000000000050673 (PMC13574484; doi:10.1097/MD.0000000000050673)
Supplement: Supplementary file 1 [file medi-105-e50673-s001.docx]

**Association between fear states and cognitive impairment: A cross-sectional observational study based on the China Health and Retirement Longitudinal Study (CHARLS)**

Xiaoyun Hui BM^a,1^, Kaikai Jiang MM^b,1^,Xiumei Xu MM^c^, Chunxuan Dong MM^c,^*, Jing Ji BM^c^

^a^Department of Rehabilitation Medicine, Wuwei People's Hospital, Wuwei, Gansu, 733099, China

^b^Tianshui Maternal and Child Health Hospital, Tianshui, Gansu, 741000, China

^c^Department of Rehabilitation Medicine, Gansu Provincial Hospital of Traditional Chinese Medicine, Lanzhou, Gansu, 730050, China

^1^The first two authors contributed equally to this work.

*Correspondence Author: Chunxuan Dong, Department of Rehabilitation Medicine, Gansu Provincial Hospital of Traditional Chinese Medicine, Lanzhou, Gansu, dongchunxuan2025@163.com

ORCID:0009-0006-8989-374X

**Short title:** Anxiety and Cognitive Decline

**List of abbreviations**

| **Abbreviations** | **Full name** |
| --- | --- |
| **CI** | **Cognitive impairment** |
| **CHARLS** | **China Health and Retirement Longitudinal study** |
| **CKD** | **Chronic kidney disease** |
| **FBG** | **Fasting blood glucose** |
| **TC** | **Total cholesterol** |
| **TG** | **Triglycerides** |
| **T2DM** | **Type 2 diabetes mellitus** |
| **vmPFC** | **Ventromedial prefrontal cortex** |
| **dACC** | **Dorsal anterior cingulate cortex** |
| **FNE** | **Fear of trait levels of negative evaluation** |
| **TBI** | **Traumatic brain injury** |
| **PTSD** | **Post-traumatic stress disorder** |
| **MMSE** | **Mini-Mental State Examination** |
| **NLRP3** | **NOD-like receptor protein 3** |
| **HPA** | **Hypothalamic pituitary adrenal** |
| **BDNF** | **Brain-derived neurotrophic factor** |

**Abstract**

**Background:** As global populations age, the prevalence of cognitive impairment continues to rise, increasing the burden on families and society. This study examined the relationship between anxiety and cognitive function.

**Methods:** Using nationally representative data from China Health and Retirement Longitudinal Study (CHARLS), this observational cohort study evaluated the association between fear states and cognitive impairment to provide objective scientific evidence on the potential causes of cognitive decline and to inform early prevention and treatment strategies.

**Results:** In this study, the prevalence of cognitive impairment was significantly higher among participants in the fearful group. After adjustment for multiple covariates, fearful state remained independently associated with cognitive impairment (odds ratio = 2.38, 95% confidence interval = 1.24–4.59, p = 9.52 × 10^−3^). Smooth curve fitting additionally confirmed this positive association.

**Conclusion:** These findings demonstrate a significant association between fear state and cognitive impairment, highlighting the need to consider emotional well-being in cognitive health assessments.

**Key words:** Cognitive dysfunction; Fear; China Health and Retirement Longitudinal Study; Anxiety; Observational study

**1. Introduction**

Cognitive impairment (CI) is a neurological condition marked by a substantial decline in cognitive ability, involving deficits in one or more key brain functions (such as memory, learning, attention, and decision-making)^[1]^. CI is prevalent among older adults, diminishing quality of life and imposing a major burden on families and society^[2]^. Demographic shifts have led to a steady global increase in the elderly population, resulting in a higher prevalence of CI^[3]^. The World Health Organization projects that by 2030, one in six individuals worldwide will be aged ≥60 years. The population aged >60 years is expected to increase from 1 billion in 2020 to 1.4 billion by 2030. Furthermore, by 2050, this group is projected to double, reaching 2.1 billion. Additionally, the population aged ≥80 years is estimated to triple, reaching 426 million between 2020 and 2050^[4]^. The primary independent risk factor for CI is advanced age^[5]^, with risk rising markedly with age. Vascular risk factors also elevate the risk of CI, and stroke increases CI risk and severity^[6]^. Moreover, individuals with chronic kidney disease face a substantially higher risk of developing CI compared with the general population^[1]^. Previous studies indicate that several variables, including age, educational attainment, diabetes duration, fasting blood glucose levels, HbA1c content, total cholesterol levels, triglyceride content, and 24-h urinary protein excretion, are independent risk factors for CI in elderly patients with type 2 diabetes mellitus^[7]^. In addition to these factors, prior research suggests that neuroinflammation plays a key role in CI pathogenesis. Systemic inflammatory responses can activate central nervous system inflammatory pathways, leading to neuronal damage, synaptic dysfunction, and neurotransmitter imbalance, thereby accelerating cognitive decline^[8,9]^. For example, a randomized controlled trial involving patients undergoing cardiac surgery showed that preoperative dexamethasone (an anti-inflammatory drug) significantly reduced the risk of early postoperative cognitive impairment while decreasing the levels of inflammatory markers, such as C-reactive protein, indicating that inflammatory responses are key mechanisms underlying CI, particularly under acute stress or trauma situations^[10]^. These findings deepen our understanding of CI’s multifactorial mechanisms and imply that emotional and stress states (such as fear) influence cognitive function by modulating inflammation. Therefore, early identification of cognitive decline–associated factors is essential for developing effective prevention and intervention strategies. This will not only improve quality of life in older adults but also reduce societal and familial burdens and better address the challenges of population aging.

Fear usually refers to an individual’s strong emotional response to perceived threats or danger. The physical and psychological features of fear include increased heart rate, sweating, heightened alertness, and related responses. Fear can also trigger anxiety and stress^[11]^. Prior neuroimaging studies in humans have primarily focused on the “fear network,” which includes key brain regions such as the ventromedial prefrontal cortex, dorsal anterior cingulate cortex, insular cortex, hippocampus, and amygdala. These regions play essential roles in modulating fear, arousal, and threat detection, and the regulation of responses to both fear and conditioned stimuli^[12]^. Evidence from attention bias research indicates that anxiety alters attentional function, although findings remain inconsistent^[13]^. Depression and anxiety are prevalent among patients with dementia. A systematic review and meta-analysis reported that the prevalence of anxiety disorders in mild, moderate, and severe dementia was 38% (95% confidence interval: 31%–45%), 41% (95% confidence interval: 31%–52%), and 37% (95% confidence interval: 8%–82%), respectively, and anxiety in vascular dementia was higher in Europe than in Asia^[14]^. These findings indicate that anxiety is closely linked to cognitive function, highlighting the need to explore interventions that reduce fear and slow cognitive decline. In addition, previous studies^[15]^ have shown that fear of negative evaluation is a risk factor for eating disorders, and higher trait levels of negative evaluation increase the likelihood of restrictive eating behaviors. CI often occurs after traumatic brain injury. Notably, memory functions are particularly vulnerable to decline during recovery from long-term injuries^[16]^. Fear-related conditions, such as post-traumatic stress disorder, may further increase the risk of aged-related cognitive decline^[17]^. Moreover, fear and avoidance of dementia symptoms in older adults can lead to adverse outcomes, including reduced quality of life, poor physical health, and memory loss, thereby accelerating cognitive decline^[18]^. Related studies^[19]^ have indicated that a strong fear of falling increases the risk of cognitive decline in older individuals. Overall, fear states appear to play an important role in the development of CI, although the precise relationship remains unclear. Therefore, the present study examines the association between fear state and CI.

The China Health and Retirement Longitudinal Study (CHARLS) is a large national development research institute sponsored by the Beijing interdisciplinary longitudinal study of the database. The survey uses a stratified, multistage probability-proportional sampling method to assess individuals aged ≥45 years across 28 provinces, including autonomous regions and municipalities directly under the central government. This survey collects data on demographics, family background, health and function, healthcare access and insurance, income, employment status, and asset ownership^[20]^. The national baseline survey was conducted in 2011 and has been followed up every 2–3 years in 2013, 2015, 2018, and 2020 to examine associations among diseases and related risk factors. Using CHARLS data, Lin et al.^[21]^ revealed that depression symptoms in middle-aged and older adults with chronic diseases are influenced by multiple factors. Additionally, Jiang et al.^[22]^ used CHARLS data to show that economic intergenerational support has no major effect on older adults’ psychological health, whereas emotional support and daily care reduce depression levels and are associated with improved mental health. Therefore, the CHARLS database provides a valuable and robust resource for research and has become an essential tool in studies of middle-aged and elderly populations.

In this context, the present study explores the association between fear state and CI using nationally representative CHARLS data. We propose the following hypothesis: among individuals aged ≥45 years, a significant positive correlation exists between self-reported fear state and CI. We test the independence and robustness of this association using multivariate statistical models, adjusting for a range of demographic and health-related covariates, to provide new evidence for understanding factors associated with CI.

2. **Materials and methods**
   2.1 **Data collection**

This observational cohort study used data from CHARLS (https://charls.pku.edu.cn/en/). In the present study, all participants were drawn from the 2018 wave, based on questionnaire data for individuals with CI aged ≥45 years (n = 19,744). First, individuals with a history of memory-related disease were excluded. In addition, participants with missing data on various variables, including sex, medical insurance, stroke, diabetes, cancer or malignant tumors, heart attack, hearing problems, health status, social activity, fear status, Mini-Mental State Examination (MMSE) scores, and education level, were excluded. Ultimately, 2,001 participants were included in the cohort analysis. The detailed participant selection process is shown in **STROBE Flow Diagram**. This study was a secondary analysis of a publicly available database and did not involve new recruitment, interventions, or biospecimen collection. The CHARLS database provides anonymized data, and ethical approval and informed consent were obtained for all primary data collection; therefore, no additional ethical approval was required for the present study. This observational cohort study was reported in accordance with the STrengthening the Reporting of OBservational studies in Epidemiology (STROBE) statement guidelines.

2.2 **CI assessment**

CI was assessed using the Chinese version of the 2018 MMSE questionnaire, which includes 24 items across 4 cognitive domains. MMSE scores ranged from 0 to 30, with lower scores indicating poorer cognitive function. Cognitive status was categorized by education level: illiteracy <17, primary school <20, secondary education or greater <24. Participants with scores <24 were classified as having CI, whereas others were categorized as non-CI (normal group)^[23]^.

**2.3 Exposure assessment**

Fear status was determined based on the questionnaire item dc014: “Have you ever felt fearful?” Participants who answered “sometimes or half the time” or “most of the time” were classified as the fearful group. Those who responded “rarely or not at all” or “not too much” served as the normal group.

**2.4 Covariates**

Additional covariates included sex (ba000_w2_3; male or female), medical insurance (ea001_w4_s12; yes or no), stroke (da007_8; yes or no), diabetes (da007_3; yes or no), cancer or malignant tumors (da007_4; yes or no), heart attack (da007_7; yes or no), hearing problems (da005_4; yes or no), health status (da002; good, fair, or bad), and activities (da056_s12; yes or no).

**2.5 Statistical analysis of participant characteristics**

All participants from the CHARLS database were divided into two groups based on the presence or absence of CI. Differences in baseline characteristics (sex, medical insurance, stroke, diabetes, cancer or malignant tumors, heart attack, hearing problems, health status, activities, and fear status) between groups were analyzed using a weighted chi-square test, with significance set at p < 0.05.

**2.6 Statistical analysis**

To assess multicollinearity between fear status and other covariates, variance inflation factors were calculated using the car package (v3.1.1; https://socialsciences.mcmaster.ca/jfox/Books/Companion/). Because some covariates were categorical with multiple levels, the generalized variance inflation factor (GVIF) and its standardized form GVIF^1/(2 × Df)^ were adopted for interpretation; values closer to 1 and well below 5 indicated no significant multicollinearity.

Within a complex sampling weighting framework, a survey design was constructed using sampling weights and clustered primary sampling units. Three weighted generalized linear models (GLMs) were fitted using the survey (v4.2.1) package (https://cran.r-project.org/web//packages/survey/survey.pdf) to evaluate the association between fear status and CI. Model I (unadjusted) included fear status only; Model II (minimally adjusted) also included sex and medical insurance; Model III was further adjusted for stroke, diabetes, cancer or malignant neoplasms, heart disease, hearing problems, health status, activities, and memory-related disorders. Adjusted odds ratios (OR) and 95% confidence intervals were calculated. The complex sampling design-based Wald F test was used to assess the overall significance of the GLMs. To evaluate calibration and model fit for Model III, weighted decile calibration curves were plotted, and calibration regression (observed outcome regressed on the logit of predicted probability) was performed. Deviance residual diagnostics were also conducted to assess model fit and identify potential outliers or influential observations. To further assess robustness, the nonlinear relationship between fear status and CI in Model III was examined using a smooth curve.

To evaluate the relative contribution of predictors, eXtreme Gradient Boosting (XGBoost; binary classification, binary:logistic) was used to assess feature importance. Data were split into training and test sets at an 80:20 ratio; fivefold stratified cross-validation was applied for hyperparameter tuning in the training set, and early stopping was used to prevent overfitting and determine the optimal number of iterations. The final model employed optimized hyperparameters: max_depth = 3, eta = 0.05, min_child_weight = 1, subsample = 0.9, colsample_bytree = 0.7, gamma = 1, with L2 regularization (lambda = 1, alpha = 0), and the optimal number of iterations (best_nrounds = 28). Feature importance rankings based on Gain and corresponding bar plots were generated to illustrate predictor contributions.

Subgroup analyses were conducted based on Model III to examine whether the association between fear status and CI varied across population subgroups. Owing to unstable model convergence caused by limited sample size, event counts, or complete separation in some subgroups, analyses were restricted to sex, activities, hearing problems, and heart attack.

All statistical analyses were performed in R (v4.2.2). Continuous variables were expressed as mean ± standard deviation. Student’s t-test was used to compare differences between two groups.

3. **Results**

**3.1 Differences in baseline characteristics**

After stratifying participants by CI status, they were categorized into CI and normal groups. As shown in **Table 1**, fear status showed a significant association with CI (p = 0.035). Specifically, 14 participants were both fearful and had CI, 110 were fearful without showing CI, 114 were not fearful but had CI, and 1,763 were neither fearful nor showed CI (**Table 1**). Additionally, medical insurance and activities differed significantly between participants with and without CI (p < 0.05). Results from the multicollinearity analysis revealed that GVIF and GVIF^1/(2Df)^ values for all variables were close to 1 and well below 5, indicating no major multicollinearity (**Table 2**).

**3.2 Association between fear status and CI risk**

Based on three weighted GLMs, association analyses revealed that the relationship between fearfulness and CI was not significantly confounded by other covariates (Model I: p = 3.35 × 10^−3^, OR = 2.63, 95% confidence interval = 1.38–5.01; Model II: p = 4.24 × 10^−3^, OR = 2.58, 95% confidence interval = 1.35–4.92; Model III: p = 9.52 × 10^−3^, OR = 2.38, 95% confidence interval = 1.24–4.59; **Table 3** and **Figure 1A**). The Wald F joint test indicated that Models I, II, and III were statistically significant (p < 0.05; **Table 4**). Moreover, the calibration curve for Model III was close to the 45° reference line, with a calibration intercept ≈ 0 and calibration slope ≈ 1, indicating no evident systematic miscalibration (**Supplementary Figure 1A**). Additionally, residuals from Model III showed a typical “double-band” distribution for binary classification outcomes, with no clear trend across fitted values, suggesting no major model misfitting (**Supplementary Figure 1B**). Furthermore, smooth curve analysis showed a positive association between fearfulness and CI prevalence (**Figure 1B**).

**3.3 Importance of fear status for CI**

XGBoost analysis of covariates showed that lack of medical insurance had the highest relative importance for CI, followed by lack of activities, poor health status, and fearfulness (**Figure 2**). These findings suggest that fear status contributes substantially to CI risk.

**3.4 Subgroup analysis of the fear status–CI association**

Subgroup analyses showed that the association between fear status and CI was generally consistent across all subgroups, with no significant interaction effects (p > 0.05). Notably, participants with fearfulness who had hearing problems or a history of heart attack exhibited a higher risk of CI (OR = 13.446 and 16.558, respectively; **Figure 3**).

**4. Discussion**

This study used the CHARLS database to investigate the association between fear status and CI. The results demonstrate a significant positive association between fearfulness and CI. Specifically, individuals experiencing fear had a higher risk of CI compared with those showing no fear, and this association remained significant after adjustment for multiple covariates, including medical insurance, health status, and diabetes. These findings suggest that fear may play a key role in cognitive decline.

This study revealed significant differences in medical insurance and activities between participants with and without CI (p < 0.05). Additionally, smooth curve analysis indicated a positive association between fear and CI prevalence, indicating that fear is linked to increased CI risk. Current evidence suggests that elevated fear levels are associated with changes in synaptic proteins in the amygdala, a key region regulating fear responses and negative emotions^[24]^. Bottinelli et al.^[25]^ reported that panic attacks involve sympathetic nervous system activation and cognitive responses triggered by perceived threats. It has also been shown that baseline brain inflammatory activity, including NLRP3 in juvenile mice, is required for conditioned flexibility in the ventral hippocampus and basolateral amygdala, and that NLRP3 loss impairs synaptic transmission and promotes anxiety-like behavior and enhanced fear learning^[26]^. Activation of the NLRP3 inflammasome has been linked to the development of various central nervous system disorders. Furthermore, NLRP3-induced and caspase-1–mediated inflammation plays a crucial role in behavioral and cognitive dysfunction in chronic brain diseases^[27]^. Prior studies have shown that the heightened CI risk associated with fear may involve amygdala dysfunction and NLRP3 inflammasome activation^[28]^. Overall, these findings highlight a significant association between fear and CI, showing that heightened fear states may contribute to cognitive decline.

Social avoidance is commonly associated with social anxiety disorder, characterized by fear, anxiety, or restlessness in social situations that may lead to reduced or avoided social engagement. Fear and chronic stress activate the hypothalamic–pituitary–adrenal (HPA) axis, leading to the release of stress hormones, such as cortisol, and prolonged cortisol elevation may adversely affect brain structure and function, especially in regions related to emotion and memory, such as the hippocampus^[29]^. These mechanisms help explain how fear states affect CI.

This study revealed that the relative importance of social activities was also higher in the CI group. Social engagement may exert neuroprotective effects through several mechanisms. First, the cognitive reserve hypothesis suggests that frequent social interaction provides sustained cognitive stimulation, enhancing neural network plasticity and complexity, thereby delaying cognitive decline^[30]^. Neuroimaging studies have shown that individuals with larger social networks possess greater gray matter volume in brain regions such as the amygdala, insula, thalamus, and frontal lobe, which are closely associated with memory, decision-making, and emotional regulation^[30]^. Second, from a neuroendocrine perspective, social isolation activates the HPA axis, leading to chronically elevated cortisol levels. Prolonged cortisol exposure can damage limbic structures, such as the hippocampus, and accelerate cognitive decline^[31,32]^. Furthermore, reduced social activity is associated with increased neuroinflammation; specifically, NLRP3 inflammasome activation may mediate social isolation–related cognitive impairment^[33]^. Third, behavioral and psychological pathways indicate that social isolation is associated with depression, reduced physical activity, and accumulation of cardiovascular risk factors, which together collectively contribute to cognitive impairment^[34]^. Notably, a large-scale cohort study by Shen et al.^[35]^ using UK Biobank data reported that social isolation increases dementia risk by 26%, independent of genetic risk. Therefore, promoting social participation among older adults may serve as an important strategy for preventing CI.

**4.1 Limitations and future research**

Although our findings show a significant association between fear status and CI, the study has several limitations. First, the exposure variable “fear state” was measured using a single self-report item (dc014) and dichotomized into fear and non-fear categories. This approach did not use standardized, multidimensional psychological scales (such as the GAD-7 or STAI) and may not fully capture the frequency, intensity, or content of fear, potentially introducing measurement error and misclassification bias. Second, owing to its cross-sectional design, this study could not determine the temporal sequence or causal relationship between fear status and CI. Third, although the model was adjusted for multiple covariates, it did not account for important variables such as depressive symptoms, socioeconomic status, smoking status, alcohol consumption, and sleep quality, potentially resulting in residual confounding. Finally, the biological mechanisms linking fear status and CI remain unclear.

To address these limitations, future research should proceed in several directions. First, standardized multidimensional scales should be used to measure fear or continuous anxiety and assess severity and subtypes more accurately. Second, longitudinal cohort studies or intervention trials should be conducted to clarify temporal relationships and causality between fear status and CI. Additionally, a broader range of covariates should be incorporated to improve the robustness and generalizability of findings. Finally, integrating neuroimaging, biochemical markers (e.g., inflammatory indicators and HPA axis–related measures), and animal models may help elucidate the biological mechanisms underlying the association between fear status and CI. These improvements may provide stronger and more comprehensive evidence on the relationship between fear and CI.

**5. Conclusions**

This study identified a significant association between fear and CI. Fear states may exacerbate CI and potentially contribute to a negative feedback cycle. However, temporal relationships and causality require further investigation in longitudinal or interventional studies. Therefore, clinicians managing CI should consider assessing emotional states, and future research should clarify the direction of this association.

**Declarations**

**Ethical Statement:**

**Ethical approval and consent to participate**

Not applicable. This study was a secondary analysis of publicly available anonymized data from the CHARLS database. No additional ethical approval was required for this observational study.

**Availability of data and materials**

The datasets analyzed during the current study are available in the [CHARLS] repository, [https://charls.pku.edu.cn/en/].

**Authors' contributions**

X.H. and K.J. and S.M. and X.X. and C.D. and J.J.; methodology, X.H.; software, K.J.; validation, X.H.; formal analysis, K.J; investigation, X.H.; resources, K.J.; data curation, X.H.; writing—original draft preparation, K.J.; writing—review and editing, S.M.; visualization, X.X.; supervision, C.D.; project administration, J.J.; funding acquisition, C.D. and J.J. All authors read and approved the final manuscript.

**Acknowledgements**

This work was supported by the study based on HIF-1a regulation of iron death of hippocampal neurons, to explore the effect and mechanism of brain-buxing ointment in improving vascular dementia [grant number GZKZ-2024-1]; and the 2022 construction project of inheritance studio of Sun Qibin, national famous old Chinese medicine expert [grant number Chinese Medicine Education Letter [2022] No. 75]. The authors declare that there are no conflicts of interest. Thanks to Bioinformatics Analysis Platform of Jining Medical University for data analysis support.

**References:**

[1] Drew DA, Weiner DE, Sarnak MJ. Cognitive Impairment in CKD: Pathophysiology, Management, and Prevention. Am J Kidney Dis. 2019;74(6):782-790. doi:10.1053/j.ajkd.2019.05.017
[2] Xia ZD, Ma RX, Wen JF, et al. Pathogenesis, Animal Models, and Drug Discovery of Alzheimer's Disease. J Alzheimers Dis. 2023;94(4):1265-1301. doi:10.3233/JAD-230326
[3] Bai W, Chen P, Cai H, et al. Worldwide prevalence of mild cognitive impairment among community dwellers aged 50 years and older: a meta-analysis and systematic review of epidemiology studies. Age Ageing. 2022;51(8):afac173. doi:10.1093/ageing/afac173
[4] Sarmiento Buitrago AF, Cerón Perdomo D, Mayorga Bogota MA. Association between cognitive impairment and socioeconomic and sociodemographic factors in Colombian older adults. Rev Colomb Psiquiatr (Engl Ed). 2024;53(2):134-141. doi:10.1016/j.rcpeng.2022.02.005
[5] Dominguez LJ, Veronese N, Vernuccio L, et al. Nutrition, Physical Activity, and Other Lifestyle Factors in the Prevention of Cognitive Decline and Dementia. Nutrients. 2021;13(11):4080. Published 2021 Nov 15. doi:10.3390/nu13114080
[6] Rost NS, Brodtmann A, Pase MP, et al. Post-Stroke Cognitive Impairment and Dementia. Circ Res. 2022;130(8):1252-1271. doi:10.1161/CIRCRESAHA.122.319951
[7] Sun L, Diao X, Gang X, et al. Risk Factors for Cognitive Impairment in Patients with Type 2 Diabetes. J Diabetes Res. 2020;2020:4591938. Published 2020 Apr 23. doi:10.1155/2020/4591938
[8] Xu Y, Yang Y, Chen X, et al. NLRP3 inflammasome in cognitive impairment and pharmacological properties of its inhibitors. Transl Neurodegener. 2023;12(1):49. doi:10.1186/s40035-023-00381-x
[9] Wu X, Gao Y, Shi C, et al. Complement C1q drives microglia-dependent synaptic loss and cognitive impairments in a mouse model of lipopolysaccharide-induced neuroinflammation. Neuropharmacology. 2023;237:109646. doi:10.1016/j.neuropharm.2023.109646
[10] Glumac S, Kardum G, Sodic L, et al. Effects of dexamethasone on early cognitive decline after cardiac surgery: a randomised controlled trial. Eur J Anaesthesiol. 2017;34(11):776-784. doi:10.1097/EJA.0000000000000647
[11] Chand SP, Marwaha R, Bender RM. Anxiety (nursing). 2021.
[12] Ressler KJ. Translating across circuits and genetics toward progress in fear- and anxiety-related disorders. Am J Psychiatry. 2020;177(3):214-222. doi:10.1176/appi.ajp.2020.20010055
[13] Gupta RS, Kujawa A, Vago DR. The neural chronometry of threat-related attentional bias: event-related potential (ERP) evidence for early and late stages of selective attentional processing. Int J Psychophysiol. 2019;146:20-42. doi:10.1016/j.ijpsycho.2019.08.006
[14] Leung DKY, Chan WC, Spector A, et al. Prevalence of depression, anxiety, and apathy symptoms across dementia stages: a systematic review and meta-analysis. Int J Geriatr Psychiatry. 2021;36(9):1330-1344. doi:10.1002/gps.5556
[15] Mancuso CJ, Spoor SP, Kambanis PE, et al. Conditional effects of state fear of negative evaluation on restrictive eating behaviors. Eat Behav. 2022;47:101676. doi:10.1016/j.eatbeh.2022.101676
[16] Fitzgerald J, Houle S, Cotter C, et al. Lateral fluid percussion injury causes sex-specific deficits in anterograde but not retrograde memory. Front Behav Neurosci. 2022;16:806598. doi:10.3389/fnbeh.2022.806598
[17] Hernandez CM, Jackson NL, Hernandez AR, et al. Impairments in fear extinction memory and basolateral amygdala plasticity in the TgF344-AD rat model of Alzheimer's disease are distinct from nonpathological aging. eNeuro. 2022;9(3). doi:10.1523/ENEURO.0181-22.2022
[18] O'Loughlin P, Pavithra P, Regan J, et al. A randomized controlled trial investigating the feasibility of a low-intensity psychological intervention for fear of memory loss and quality of life in older adults: protocol for the reducing fear and avoidance of memory loss (REFRAME) study. JMIR Res Protoc. 2021;10(7):e30514. doi:10.2196/30514
[19] Noh HM, Roh YK, Song HJ, et al. Severe fear of falling is associated with cognitive decline in older adults: a 3-year prospective study. J Am Med Dir Assoc. 2019;20(12):1540-1547. doi:10.1016/j.jamda.2019.06.008
[20] Zhao Y, Hu Y, Smith JP, et al. Cohort profile: the China health and retirement longitudinal study (CHARLS). Int J Epidemiol. 2014;43(1):61-68. doi:10.1093/ije/dys203
[21] Lin W, Zhang D, Wang YM, et al. Analysis of depression status and influencing factors in middle-aged and elderly patients with chronic diseases. Front Psychol. 2024;15:1308397. doi:10.3389/fpsyg.2024.1308397
[22] Jiang Z, Liu H, Deng J, et al. Influence of intergenerational support on the mental health of older people in China. PLoS One. 2024;19(4):e0299986. doi:10.1371/journal.pone.0299986
[23] Chen Q, Zhou T, Zhang C, et al. Exploring relevant factors of cognitive impairment in the elderly Chinese population using Lasso regression and Bayesian networks. Heliyon. 2024;10(5). doi:10.1016/j.heliyon.2024.e27069
[24] Hoffman AN, Watson S, Chavda N, et al. Increased fear generalization and amygdala AMPA receptor proteins in chronic traumatic brain injury. J Neurotrauma. 2022;39(21-22):1561-1574. doi:10.1089/neu.2022.0119
[25] Bottinelli F, Delvecchio G, Moltrasio C, Ferro A, Diwadkar VA, Brambilla P. Facial emotion recognition in panic disorder: a mini-review of behavioural studies. J Affect Disord. 2021;282:173-178. doi:10.1016/j.jad.2020.12.064
[26] Komleva YK, Lopatina OL, Gorina IV, et al. NLRP3 deficiency-induced hippocampal dysfunction and anxiety-like behavior in mice. Brain Res. 2021;1752:147220. doi:10.1016/j.brainres.2020.147220
[27] Liang T, Zhang Y, Wu S, et al. The role of NLRP3 inflammasome in Alzheimer's disease and potential therapeutic targets. Front Pharmacol. 2022;13:845185. doi:10.3389/fphar.2022.845185
[28] Komleva YK, Lopatina OL, Gorina IV, et al. NLRP3 deficiency-induced hippocampal dysfunction and anxiety-like behavior in mice. Brain Res. 2021;1752:147220. doi:10.1016/j.brainres.2020.147220
[29] Salihu SA, Ghafari H, Ahmadimanesh M, et al. Glatiramer acetate attenuates depressive/anxiety-like behaviors and cognitive deficits induced by post-weaning social isolation in male mice. Psychopharmacology. 2021;238:2121-2132. doi:10.1007/s00213-021-05836-5
[30] Arshad F, Boopalan D, Arora S, et al. Association between social networking and dementia: a systematic review of observational studies. Neuroscience. 2025;576:138-148. doi:10.1016/j.neuroscience.2025.04.019
[31] Matthews T, Rasmussen LJH, Ambler A, et al. Social isolation, loneliness, and inflammation: a multi-cohort investigation in early and mid-adulthood. Brain Behav Immun. 2024;115:727-736. doi:10.1016/j.bbi.2023.11.022
[32] Haucke M, Golde S, Saft S, et al. The effects of momentary loneliness and COVID-19 stressors on hypothalamic-pituitary-adrenal (HPA) axis functioning: a lockdown stage changes the association between loneliness and salivary cortisol. Psychoneuroendocrinology. 2022;145:105894. doi:10.1016/j.psyneuen.2022.105894
[33] Maneemai O, Maneemai K. Social isolation and loneliness as modifiable risk factors for dementia: evidence-based interventions and public health implications. Adv Neurol. 2025:025220067
[34] Elovainio M, Lahti J, Pirinen M, et al. Association of social isolation, loneliness and genetic risk with incidence of dementia: UK Biobank cohort study. BMJ Open. 2022;12(2):e053936. doi:10.1136/bmjopen-2021-053936
[35] Shen C, Rolls ET, Cheng W, et al. Associations of social isolation and loneliness with later dementia. Neurology. 2022;99(2):e164-e175. doi:10.1212/WNL.0000000000200583

**Figure legend**

**Figure 1**: Association between fearful state and the risk of CI, (A) Forest plot of the association between fearful state and CI risk based on the Model III. The red dots in the figure represent the OR of each variable, and the horizontal lines represent the 95% confidence intervals. The dotted line represents the invalid line with OR = 1. The OR (95% CI) and Wald test p value are given on the right side. (B) Smooth curve fitting of the correlation between fearful state and the predicted prevalence of CI. The horizontal axis represents the Fearful state (0 = not fearful, 1 = fearful; continuous scales are used to show the trend of smooth fitting), and the vertical axis represents the probability of CI predicted by the model.

**Figure 2**: The results of covariates calculation by XGBoost algorithm.

**Figure 3**: Subgroup analysis of the association between fearful state and CI risk in the CHARLS cohort (Model III).

**Supplementary Figure 1:** Calibration and fitting evaluation of the Model III. (A) The weighted quantile array calibration curve of Model III. Divide the samples into 10 groups (deciles) based on the predicted probabilities. Each point represents the weighted average predicted probability and weighted observed probability of that group, with solid lines connecting the points of each group. The dotted line is the ideal calibration reference line (y = x). The closer the point is to the dotted line, the better the calibration of the model. (B) The bias residual diagnostic diagram of Model III. The horizontal axis represents the fitting probability (fitted values) of model 3 in the analyzed samples, the vertical axis represents the corresponding deviation residuals, and the dotted line indicates the reference line with a residual of 0.
